# A Systematic Review and Meta-Analysis of the Management of Gallstone Cholecystitis and Common Biliary Duct Stones to Reduce the Incidence of Complications in Elderly Patients

**DOI:** 10.7759/cureus.63115

**Published:** 2024-06-25

**Authors:** Premjithlal Bhaskaran, Christie Swaminathan, Dominika Krasicka, James A Gilbert, India P Bhaskaran, Mansoor Khan

**Affiliations:** 1 Department of Cardiothoracic Surgery, Hammersmith Hospital, Imperial College London, London, GBR; 2 Department of Gastrointestinal Surgery, University Hospitals Sussex NHS Foundation Trust, Brighton, GBR; 3 Department of Surgery, The New Foscote Hospital, Banbury, GBR; 4 Department of Digestive Disease and General Surgery, Royal Sussex County Hospital, Brighton, GBR; 5 Department of Cardiovascular Surgery, Imperial College London, London, GBR; 6 Department of General Surgery, University Hospitals Sussex NHS Foundation Trust, Brighton, GBR

**Keywords:** open cholecystectomy, cholectst, endoscopy ercp, laparoscopic cholecystectomy, conservative management

## Abstract

As the age increases particularly above the age of 50 years, there is a significantly higher risk of developing gallstone-related complications especially cholecystitis and common bile duct stones with its associated consequences. Complications that arise after surgical operations for cholecystitis have been reported to have negative impacts on senior patients. These effects include a higher rate of complications, a longer hospital stay, higher expenditures, and decreased patient satisfaction. Therefore, finding the most effective treatment for cholecystitis in older patients is still a challenge. The aim of the study was carried out in order to identify many approaches that can be taken in the treatment of cholecystitis and stones in the common bile duct in older patients. A search was conducted through Medline (PubMed), EMBASE, ProQuest, and Cochrane using relevant Medical Subject Heading (MeSH) terms and keywords (elderly, age over 50, cholecystitis, bile duct stones, cholecystectomy, ERCP, surgical, conservative management, and open). The searches were limited to studies on elderly individuals over 50 who had cholecystectomy and endoscopic retrograde cholangiopancreatography between January 2000 and December 2022. The meta-analysis used the Mantel-Haenszel odds ratio (MHOR) and 95% confidence interval (CI). Aries Systems Corporation's Editorial Manager® (Aries Systems Corporation, North Andover, USA) and ProduXion Manager® (Aries Systems Corporation, North Andover, USA) facilitated the study. Out of 102 citations, 39 studies were selected for further study. After that, 18 studies were eliminated, leaving 21 for meta-analysis. The study found a protective risk of cholecystitis in cholecystectomy patients (MHOR = 0.16; 95%, CI = 0.10 to 0.25; p 0.001). Developing cholecystitis was substantially lower in early cholecystectomy patients (MHOR = 0.16; 95%, CI = 0.10 to 0.25; p 0.001). There was no significant difference in cholecystitis risk between open and laparoscopic surgery (MHOR = 0.65; 95%, CI = 0.41 to 1.04; p 0.07). Cholecystectomy performed at an earlier stage protects elderly patients from developing recurrent cholecystitis. In contrast to late cholecystitis, in which the patient would experience several attacks of cholecystitis, early cholecystectomy protects against the recurrence of the condition.

## Introduction and background

As age increases so does the risk of developing gallstones and their associated complications such as cholecystitis. There is a higher prevalence of cholecystitis and the complications that occur with it in the more elderly population. In around 20% of cases that require cholecystectomy, patients also have stones in their common bile duct (CBD) [1]. Even small stones have the potential to induce complications such as jaundice, cholangitis, pancreatitis, or hepatic abscesses. As a result, it is recommended that procedures be used to remove CBD stones. Endoscopic retrograde cholangiopancreatography (ERCP) and laparoscopic cholecystectomy (LC) are the two procedures that are most frequently used to treat CBD stones in senior patients. For the removal of CBD stones, a method that combines laparoscopic cholangioscopy and intraoperative laparoscopic exploration of the bile duct has been demonstrated to be both safe and successful [1,2]. Before performing cholecystectomy or ERCP on patients who have severe gallstone pancreatitis, it is standard clinical practice to wait until the patient has recovered from the initial inflammation and then execute the procedure in question [2].

Depending on when they are discovered - before, during, or after cholecystectomy - CBD stones require a variety of treatment approaches to be managed effectively. The most important considerations in selecting a therapy are the surgeon's level of skill in laparoscopy, the quantity and size of stones, and the level of patient satisfaction. Laparoscopic CBD exploration (transcholedochal or transcystic), ERCP with or without endoscopic biliary sphincterotomy, or laparotomy with CBD exploration (by T-tube, C-tube insertion, or primary closure) are procedures that are utilised most frequently in the management of CBD stones [2]. LC is being conducted more frequently on an ageing population, despite the fact that the dangers are inadequately quantified [3]. The purpose of this study was to examine the existing evidence in order to estimate the postoperative risk after cholecystectomy and to identify the various strategies for the management of cholecystitis and stones in the CBD in senior patients. Specifically, the review will focus on instances involving people who are over the age of 50.

Aim

The purpose of this study was to determine the various therapy options available for cholecystitis and stones in the CBD in elderly people.

Materials and methods

Using a combination of the pertinent Medical Subject Heading (MeSH) terms and the keywords (elderly, age more than 50 years, cholecystitis, bile duct stones, cholecystectomy, ERCP, surgical, conservative management, and open cholecystectomy), a search was conducted through the databases of Medline (PubMed), EMBASE, ProQuest, and Cochrane. Within the scope of the search in the Cochrane database, the word "clinical trial" served as the filter. The time frame for the searches was limited between January 2000 and December 2022, and only papers published in English were considered. Selected papers were limited to studies that were conducted on patients above the age of 50 and who had undergone cholecystectomy and ERCP. The Mantel-Haenszel odds ratio (MHOR) along with its 95% confidence interval (CI) were obtained for the meta-analysis.

The titles and abstracts of the cited studies were reviewed, and complete reports were only obtained if the studies were clinical research and addressed the treatment of cholecystitis and stones in the CBD in patients older than 50 years.

Inclusion Criteria

Randomised controlled trials (RCTs), cohort studies, and comparative studies that attempted to address the management of cholecystitis and stones in the CBD were the types of studies that were considered eligible for inclusion.

Exclusion Criteria

Studies were deemed ineligible for review if the relevant outcome measure (the management of cholecystitis and stones in the CBD) was either not reported at all or could not be extracted or calculated from the data that was made available.

Search Strategy

The treatment of cholecystitis and stones in the CBD were used as the screening criteria for cases of cholecystitis in patients older than 50 years. During the second step, complete papers of all the research that met the screening criteria were collected. Each of these papers was evaluated using a set of selection criteria, and only valid studies were included in the analysis of the collected data.

Data Collection Methods

In several databases, the terms "elderly, age more than 50 years, cholecystitis, bile duct stones, cholecystectomy, ERCP, surgical, conservative management, and open cholecystectomy" were input, and a search was carried out by year. It was determined whether the content of the studies could be deduced from the titles or abstracts of the research, and then the entire manuscripts of the studies were obtained. All selected publications were downloaded, read and evaluated based on the eligibility criteria, and a list of the studies that were chosen was compiled. They underwent additional testing for inclusion, and pertinent data was gleaned from the results.

Quality Assessment

The methodological quality of each of the studies that were included in the meta-analysis was evaluated with the Cochrane risk of bias assessment tool and the Joanna Briggs Institute (JBI) checklist for cohort studies and comparative studies [4-6]. Both of these tools were utilised. The response was recorded as either "yes" or "no" for each item, and a credit point of "one" was assigned for "yes," while "zero" credit points were assigned for "no." Complete tallies of every one of the points were compiled. A higher count suggests a more thorough appraisal (Table 1).

**Table 1:** Critical appraisal of the studies included in the meta-analysis RCT: randomised controlled trial

Authors	Design	Sample size	Appraisal score
Boo et al. [7]	RCT	33	6/7
Catena et al. [8]	RCT	144	5/7
Chau et al. [9]	Cohort	73	9/12
da Costa et al. [10]	RCT	235	6/7
Gutt et al. [11]	RCT	542	4/7
Heo et al. [12]	Cohort	120	9/12
Jain et al. [13]	Cohort	97	10/12
James et al. [14]	Cohort	178	8/12
Jee et al. [15]	RCT	49	5/7
Johansson et al. [16]	RCT	70	5/7
Lau et al. [17]	Cohort	151	9/12
Nakai et al. [18]	Cohort	294	9/12
Noel et al. [19]	RCT	56	4/7
Pessaux et al. [20]	Comparative	139	8/10
Reinders et al. [21]	Cohort	75	10/12
Ridtitid et al. [22]	Cohort	79	9/12
Schmidt et al. [23]	Cohort	54	10/12
Schmidt et al. [24]	RCT	104	6/7
Sousa et al. [25]	Cohort	131	10/12
Tsujino et al. [26]	Cohort	194	9/12
Zargar et al. [27]	RCT	131	5/7

Data Analysis

Utilising the fixed effect model allowed for the computation of the MHOR as well as the 95% CI. The Chi-square statistic and the I2 statistic were utilised in order to test for the presence of heterogeneity [6]. For the purpose of data analytics, the Review Manager Software (RevMan 5, developed by the Cochrane cooperation in Oxford, England) was utilised.

## Review

Results

**Figure 1:**
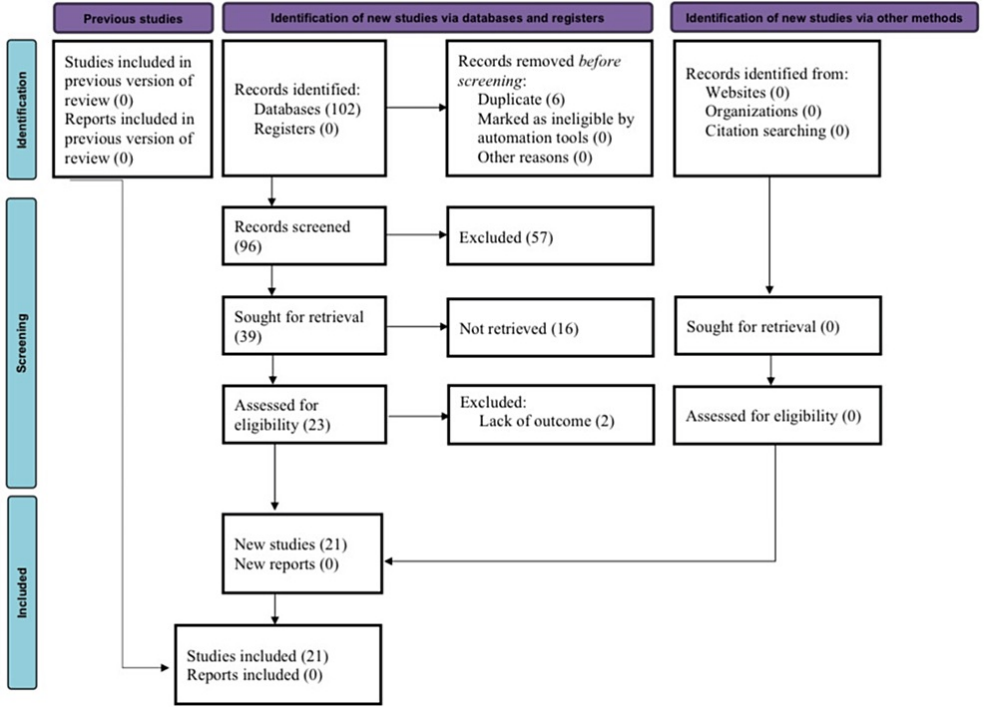
PRISMA flow diagram PRISMA: Preferred Reporting Items for Systematic Reviews and Meta-Analyses

The initial search turned up a total of 102 citations, out of which 39 studies were retrieved for further examination. Sixteen investigations were disregarded as insufficient. During the second phase, a meta-analysis was performed on 21 of the 23 studies that were still in existence (Figure 1) [7-27].

**Figure 2:**
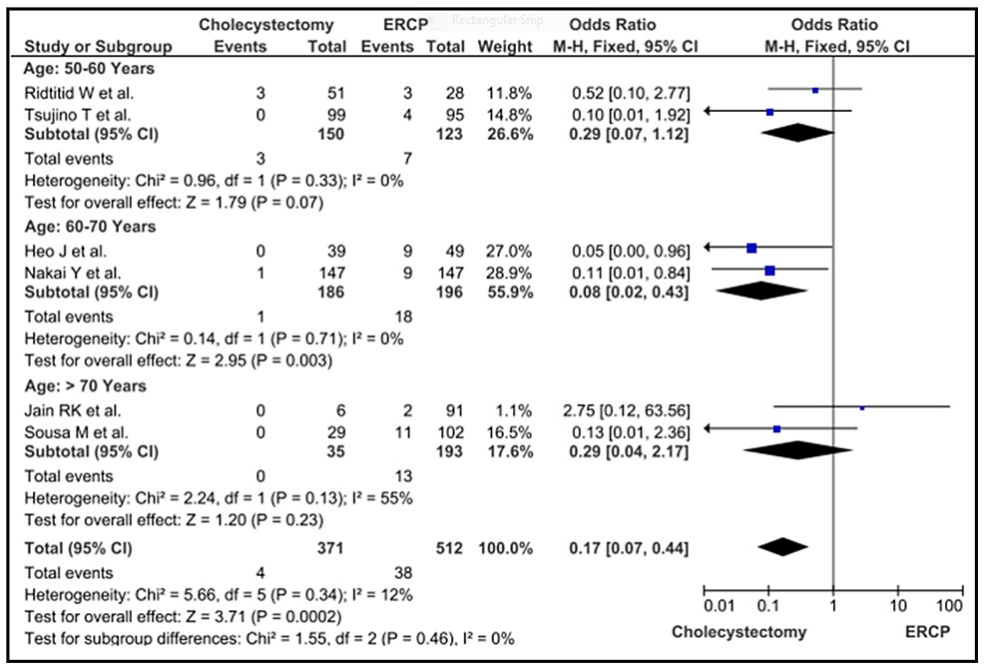
Cholecystectomy vs ERCP ERCP: endoscopic retrograde cholangiopancreatography Heo et al. [12], Jain et al. [13], Nakai et al. [18], Ridtitid et al. [22], Sousa et al. [25] and Tsujino et al. [26]

A total of 2949 samples were submitted by the studies that were chosen for the meta-analysis (n = 21). Cases that had cholecystectomy, displayed a protective risk (MHOR = 0.08; 95% CI = 0.02 to 0.43; p = 0.003) of cholecystitis when compared to ERCP cases. This was seen among patients in the age category of 60-70 years. There was no significant difference in the risk between people in the age group 50-60 years (MHOR = 0.29; 95% CI = 0.07 to 1.12; p = 0.07) and people older than 70 years (MHOR = 0.29; 95%, CI = 0.04 to 2.17; p = 0.23). However, when cholecystectomy patients were compared to ERCP patients of all ages, cholecystectomy patients were shown to have a significantly lower risk of mortality (MHOR = 0.17; 95% CI = 0.07 to 0.44; p = 0.001) (Figure 2).

**Figure 3:**
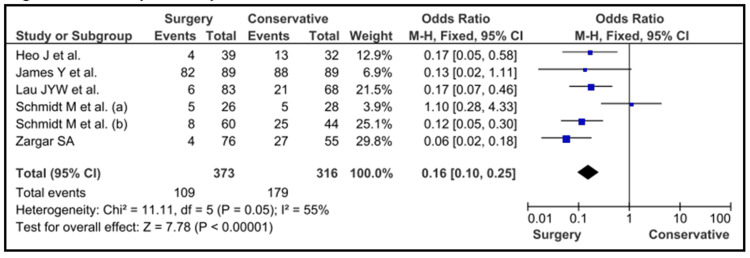
Cholecystectomy vs conservative management Heo et al. [12], James et al. [14], Lau et al. [17], Schmidt et al. [23], Schmidt et al. [24] and Zargar et al. [27]

Those who were treated with cholecystectomy displayed a protective risk (MHOR = 0.16; 95% CI = 0.10 to 0.25; p < 0.001) as compared to those who were treated with conservative therapy (Figure 3).

**Figure 4:**
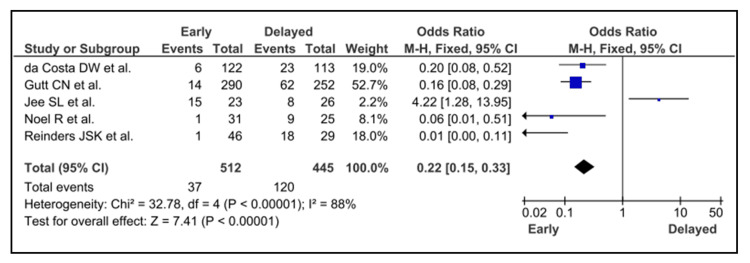
Early vs delayed cholecystectomy da Costa et al. [10], Gutt et al. [11], Jee et al. [15], Noel et al. [19] and Reinders et al. [21]

In cases of early cholecystectomy, there was a protective risk of cholecystitis (MHOR = 0.16; 95% CI = 0.10 to 0.25; p < 0.001), as compared to delayed (Figure 4).

**Figure 5:**
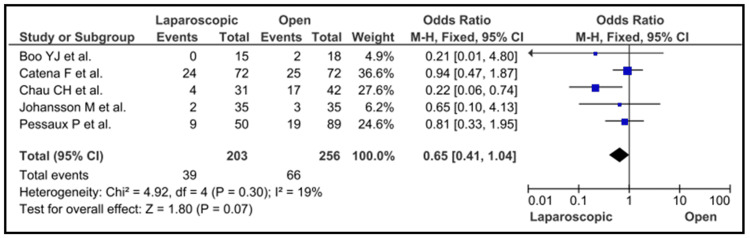
Laparoscopic vs open cholecystectomy Boo et al. [7], Catena et al. [8], Chau et al. [9], Johansson et al. [16] and Pessaux et al. [20]

In addition, there was no significant difference in the risk of cholecystitis between cases that were treated by LC and those who were treated with open cholecystectomy (MHOR = 0.65; 95% CI = 0.41 to 1.04; p < 0.07) (Figure 5).

Discussions

Cholecystectomy gallstones are frequently accompanied by clinical symptoms of severe cholecystitis, including systemic sequelae and the potential for further complications [28]. Percutaneous drainage (cholecystostomy) is an alternative treatment to urgent cholecystectomy and it can be performed safely and effectively despite the necessity of systemic support and antimicrobial therapy. It eliminates the risks associated with general anaesthesia, particularly in a multimorbid aging population. The procedure can also be carried out either as a preliminary bridging step before a planned surgical procedure or as the final treatment for certain patients including those felt to be too high risk for surgery at any stage. It is, however, still not clearly established if an emergency cholecystectomy is a better choice of treatment compared with percutaneous drainage. There is a clinical assumption that in older, multimorbid patients, cholecystostomy is less invasive and therefore the better form of treatment for these patients. This is also compounded by the severe lack of resources and operating theatre facilities to undertake an emergency LC in a timely fashion after an acute presentation.

For the treatment of older people who are experiencing acute cholecystitis, one of the available options is a conservative management approach, which is a non-invasive strategy [13,28]. On the other hand, because the source of the infection is not under control, there is an increased risk of biliary sepsis. Patients who are elderly require additional care during the postoperative period because they are more likely to suffer from pulmonary problems, ulceration, undernutrition, disorientation, urinary tract infections, and functional decline. This necessitates greater attention being paid to these patients.

Limitations

The most significant drawbacks of this study are an analysis of the retrospective features of the older patients and the absence of a reference group with similar demographics. It is possible that longitudinal advantages of the various approaches for the therapy of cholecystitis and stones in the CBD in older patients might be provided by well-structured and homogenous prospective data with a follow-up period of 24 months.

## Conclusions

Cholecystitis can be treated in elderly patients using a variety of procedures, including ERCP in CBD stones, LC, open cholecystectomy, early or delayed cholecystectomy, and conservative maintenance. Cholecystectomy, when performed at an earlier stage, confers protection against the development of recurrent cholecystitis in senior patients. Early cholecystectomy, on the other hand, guards against the recurrence of the ailment, in contrast to late cholecystitis, in which the patient would undergo many attacks of cholecystitis.
